# Low prevalence of *Leishmania donovani* infection among the blood donors in kala-azar endemic areas of Bangladesh

**DOI:** 10.1186/1471-2334-13-62

**Published:** 2013-02-02

**Authors:** M Mamun Huda, Shikha Rudra, Debashis Ghosh, Khondaker Rifat Hasan Bhaskar, Rajib Chowdhury, Aditya Prasad Dash, Sujit Kumar Bhattacharya, Rashidul Haque, Dinesh Mondal

**Affiliations:** 1 Centre for Communicable Disease, icddr,b, Mohakhali, Dhaka 1212, Bangladesh; 2 Blood Transfusion Department, Mymensingh Medical College, Mymensingh, Bangladesh; 3 Department of Medical Entomology, National Institution of Preventive and Social Medicine (NIPSOM), Dhaka 1212, Bangladesh; 4 Vector-Borne and Neglected Tropical Diseases Control, Regional Office for South-East Asia, World Health Organization, New Delhi 110002, India; 5 Centre for Nutrition and Food Security, icddr,b, Mohakhali, Dhaka 1212, Bangladesh

**Keywords:** Visceral leishmaniasis, Kala-azar, Blood donors, Transfusion, *Leishmania donovani*, Bangladesh

## Abstract

**Background:**

Visceral leishmaniasis (VL) is a major public health problem in Bangladesh with the highest disease burden in the Mymensingh District. The disease is transmitted by sand fly bites, but it may also be transmitted through blood transfusions. No information is available about the prevalence of Leishmania infection among blood donors in Bangladesh; therefore we aimed to investigate this question.

**Methods:**

The study was carried out in the Blood Transfusion Department of Mymensingh Medical College Hospital. One thousand one hundred and ninety five adult healthy blood donors attending in this department were enrolled in the study from August 2010 to April 2011. After obtaining written consent, socio-demographic data and a detailed health history were collected. The medical officer in the unit performed a complete physical examination to exclude any acute or chronic diseases, which was followed by sero-diagnosis for exposure to Leishmania by rK39 strip test using finger prick blood. Blood donors with a positive rK39 strip test underwent a PCR test for detection of leishmania DNA in their peripheral blood buffy coat.

**Results:**

Eighty two percent of enrolled blood donors were male (n=985) and 18% (n=210) were female. The mean age of blood donors was 27 years (SD, 7.95 years). The majority of donors were literate and had mid-to-higher socioeconomic condition reflected by household conditions reported by the subject. Only 2.6% had a family member with VL in the past. Three blood donors were positive for leishmania infection by rK39 strip test (0.3%, 95%CI, 0.05%-0.73%). None of these 3 had active leishmania infection as demonstrated by PCR analysis. During six months of follow up, neither rK39 positive (n=3) nor rK39 negative (n=1192) donors developed VL.

**Conclusion:**

The prevalence of *Leishmania donovani* infection among blood donors attending the Blood Transfusion Department of Mymensingh Medical College Hospital was very low. Therefore the chance for transmission of VL through blood transfusion is negligible. We believe that the National VL Elimination Program does not need set up routine screening for *Leishmania donovani* infection in blood transfusion departments located in VL endemic areas of Bangladesh.

## Background

Human visceral leishmaniasis (VL) / kala-azar (KA) is a severe chronic disease caused by parasites of the *Leishmania donovani* complex. The disease is lethal if left untreated and affects approximately half a million new patients annually worldwide, with 60% of new cases on the Indian sub-continent [1]. The disease is highly clustered geographically, and Mymensingh District is the most highly-endemic district out of 45 affected districts in Bangladesh [2,3]. *Leishmania donovani* is the only species that causes VL on the Indian sub-continent, including Bangladesh. The female sand fly *Phlebotomus argentipes* is only vector responsible for transmitting the disease in this region, and humans are the only reservoir [1-3]. Recent advances in leishmaniasis research have lead to the discovery of a very simple way to diagnose VL using an immunochromatographic test (ICT), which detects antibody against the rK39 antigen of the Leishmania parasite, and home-based treatment of VL is now possible with the oral drug Miltefosine. The unique epidemiologic characteristics of VL on the Indian sub-continent, along with these recent advances in diagnosis and treatment, make it possible to control or even eliminate this disease [4]. These facts inspired the Health Ministers from Bangladesh, India and Nepal to sign a Memorandum of Understanding in 2005 to eliminate VL from the sub-continent by 2015 [5]. The goal of the elimination program was to reduce VL cases below 1 per 10,000 people in the VL endemic areas [5] through 1) active case detection and proper management of cases with VL and Post-Kala-azar Dermal Leishmaniasis (PKDL); 2) interruption of disease transmission through integrated vector management strategies; and 3) social mobilization [4,5].

Recent literature has suggested that VL disease may be transmitted through blood transfusions if blood donors have active infection with the leishmania parasite. The first reported transmission of VL through blood transfusion was documented in 1948 [6], and since then there has been increasing evidence of transfusion-transmitted VL in VL endemic countries [7-13]. No information is available about VL transmission through blood transfusion in Bangladesh. Mymensingh is the most highly-endemic district for VL in Bangladesh, where more than 50% of the national burden of VL cases has been reported. The Blood Transfusion Department of Mymensingh Medical College Hospital is the only public blood transfusion unit in the district, serving patients of the medical college and those referred by 12 sub-district hospitals in Mymensingh. Usually, blood donors attending this department are relatives of patients who require blood transfusions. Since VL is clustered at the household and village level, theoretically there is a risk of VL transmission through blood transfusion if active Leishmania infection is found among blood donors. This may necessitate the introduction of routine screening for Leishmania infection among blood donors by the National VL Elimination Program. We have therefore undertaken this study to fill this knowledge gap in Bangladesh to support the National VL Elimination Program. Our study investigates the prevalence of Leishmania infection among blood donors in VL endemic areas of Bangladesh.

## Methods

### Study site and population

The study was carried out in the Blood Transfusion Department, Mymensingh Medical College Hospital, Mymensingh District from August 2010 to April 2011. During the study period, voluntary blood donors attending in the department were invited to participate in the study. On each working day, up to 10 voluntary blood donors were enrolled in the study after obtaining written informed consent and passing screening based on study inclusion and exclusion criteria. The study inclusion criteria were: age between 18–60 years old; either sex; clinically healthy; no history of VL in the past; permanent resident of Mymensingh District; and voluntary consent to participate in the study. Exclusion criteria were: age less than 18 years old or more than 60 years old; clinical evidence of acute or chronic illness; past history of Kala-azar or PKDL; presence of PKDL-like skin lesions; and refusal to consent to study participation.

### Study design and sampling

The study design was a cross-sectional survey with 6 months follow up. A trained field research assistant (FRA) consented prospective subjects and collected socio-demographic information using a structured questionnaire, then the medical officer of the transfusion unit performed a clinical assessment and enrolled study participants after checking inclusion and exclusion criteria. The medical laboratory technician of the transfusion unit performed the rK39 strip test (rK39 ICT, Kala-azar DetectR, In Bios International, Seattle, USA) using finger prick blood from the participants, as per the manufacturer’s instructions. If a study participant tested positive for Leishmania infection by the rK39 strip test, then a lab technician collected 3 ml venous blood in an EDTA tube and this was transported to the Parasitology Laboratory, icddr,b maintaining cold chain for buffy coat preparation, DNA isolation and Ln-PCR analysis (see laboratory methods). All rK39 positive blood donors were regarded as not eligible for blood donation. Since the median incubation period for developing VL is approximately four months [2], all study participants, including those with positive rK39 strip tests, were followed for six months for eventual development of VL using mobile phone contact or home visits (when possible). All participants were instructed to contact study staff if they developed a fever of more than two weeks, or experienced weight loss, skin darkening and/or abdominal enlargement within six months of enrollment.

### Laboratory methods

#### Preparation of buffy coat

Upon receipt at the laboratory, blood samples were centrifuged at 8000 rpm for 10 minutes at room temperature, and 500 μL of buffy coat was collected from the middle layer of the tube containing concentrated leukocytes. The buffy coat was kept in a 1.5-mL sterile microcentrifuge tube and preserved at −20°C for DNA extraction and PCR amplification.

#### DNA extraction from buffy coat

Buffy coat DNA was extracted for PCR using a QIAamp DNA blood mini kit (Qiagen, Hilden, Germany, Cat. no. 51106), as per the manufacturer’s instructions. The DNA was eluted in 0.2 mL of AE buffer (supplied with the Qiagen kit) after extraction. The purity of the DNA was satisfactory since the OD ratio at A260/A280 was within 1.7-1.9 for all DNA samples. Molecular-grade water was used instead of blood as an extraction control for checking for carry-over contamination in every run of DNA extraction and PCR amplification.

### PCR analysis

*Leishmania-*specific nested PCR (Ln-PCR) was performed to detect parasite DNA in peripheral buffy coat using 2 μL of extracted DNA by the method described previously [14], with primers targeting the parasite’s SSU-rRNA region [15]. For the first PCR run, we used primers R221 5^′^-GGTTCCTTTCCTGATTTACG-3^′^ and R332 5^′^- GGCCGGTAAAGGCCGAATAG-3^′^. For the second PCR, 1 μL of 1:50 dilution of the first PCR product was used as a template in the presence of 0.15 μmol/L of the *Leishmania-*specific primers R223 and R333.

### Sample size calculation

Seroprevalence by the rK39 strip test among individuals without past VL infection is 6% in the highly endemic areas of Mymensingh District, with 3% of seropositive individuals having confirmed active leishmania parasite infection by polymerase chain reaction (PCR) analysis (unpublished data). Thus to demonstrate an active infection rate of 3% among blood donors with 1% precision and 95% confidence interval (CI), a minimum sample size of 1,117 blood donors was required.

### Data analysis

Socio-demographic data and laboratory results were entered into EPI Info 3.5.1 software (CDC, Atlanta, GA) for windows. After cleaning the data and checking for duplicates we explored the descriptive statistics to interrogate the nature of the data. A 95%CI was calculated using Normal as well as Poisson distributional approaches, where applicable. We analyzed all data using the statistical software STATA 10.1 (Stata Corporation, College Station, Texas).

### Ethical statement

Blood donors were enrolled in the study after obtaining written consent. All rK39 positive blood donors were informed of their status and advised not to donate blood for the next six months. All rK39 positive blood donors were then followed for six months to see if they developed VL. The study was approved by the Research Review Committee and Ethical Review Committee of icddr,b, Dhaka, Bangladesh; and the WHO Regional Office for South-East Asia, New Delhi, Inida. The study was also approved by the Directorate General of Health Services, Government of Bangladesh.

## Results

### Blood donor characteristics

During the study period, a total of 14,367 voluntary blood donors attended the Blood Transfusion Department of Mymensingh Medical College Hospital for blood donation. Among them, 1,195 blood donors were enrolled in the study. Approximately 82% of the blood donors were male and the average (SD) age of the donors was 27.66 (7.95) years. Fifty percent of enrollees reported donating blood at least once per year. Almost all the donors were donating blood for their relatives or friends, and more than 90% were aware of Kala-azar (Table 1).

**Table 1:** Characteristics of blood donors in Mymensingh Medical College Hospital, Mymensingh (N=1195)

**Characteristics**	**n (%)**
Sex	
– Male	985 (82.4)
– Female	210 (17.6)
Mean Age in years (SD)	27.66 (7.95)
Education	
– Illiterate	80 (6.7)
– Literate	1115 (93.3)
Occupation	
– Own agriculture	77 (6.4)
– Skilled worker	43 (3.6)
– Unskilled worker	13 (1.1)
– Own business	256 (21.4)
– Job holder	246 (20.6)
– Student	432 (36.2)
– Housewife	117 (9.8)
– Others	11(0.9)
Donate blood at least once in a year	594 (49.7)
Reason for blood donation	
– Relative or friend patient	1187 (99.4)
– Registered blood donor	7 (0.6)
Heard about Kala-azar	1128 (94.4)
House type of blood donor	
– Precarious	62 (5.2)
– Non-precarious	1133 (94.8)
Mean no. of living room (SD) in the household	3.53 (1.61)
Average family size (SD)	5.7 (2.3)
Average number of bed-nets (SD) in the household	3.31 (1.41)
Use bed net previous night	997 (83.4)
Past KA patients in the household	31 (2.6)

The majority of blood donors had mid-to-high socioeconomic status, as reflected by household and living conditions (non-precarious house 94.8%; average number of rooms in the house 3.53). The majority of participants also practiced protection against mosquito/sand fly bites with 83.4% reporting they used a bed-net the previous night. The average family size was 5.7 members, and only 2.6% of participants reported having a family member with VL in the past (Table 1).

### Prevalence of Leishmania infection among blood donors

We identified only 3 individuals out of 1,195 with positive rK39 strip tests, indicating that the prevalence of Leishmania infection among these blood donors was 0.3% (n=3, 95%CI, 0.05%-0.73%). None of the positive blood donors were positive for Leishmania DNA in their peripheral blood, indicating that none suffered from active Leishmania infection (Table 2). One of the 3 positive blood donors came from a previously VL-infected household. None of the 1,195 participants developed VL during the 6 months follow-up period.

**Table 2:** Serological analysis of blood from the blood donors in Mymensingh Medical College Hospital, Mymensingh

**Serological test**	**n (%)**
rK39 test results; N=1195	
– Positive	3 (0.3)
– Negative	1192 (99.7)
PCR test done; N=3	3 (100.0)
PCR results; N=3	
– Positive	0 (0.0)
– Negative	3 (100.0)

## Discussion

This is the first study to investigate the prevalence of Leishmania infection among blood donors in Bangladesh, and the major finding of the study is that the prevalence of active Leishmania infection among blood donors in Mymensingh District is very low. Our study results suggest there is a negligible risk of transmission of Leishmania infection through donated blood from healthy voluntary blood donors in Mymensingh. This is a welcome news for the National VL Elimination Program, because our study would suggest that the introduction of routine screening for Leishmania infection among blood donors in the VL endemic districts of Bangladesh is unnecessary.

VL is well known as a disease of the poorest of the poor. Therefore the low prevalence of Leishmania infection among blood donors in our study may be attributable to their higher socio-economic status, as demonstrated by better housing conditions, the number of rooms and bed-nets in the household and bed-net usage. It is also possible that the prevalence rate found in our study was underestimated due to the use of rapid (rK39) tests for detection of *Leishmania donovani* infection. The use of the Direct Agglutation Test (DAT) has been recommended for detection of VL infection [16]; however, many studies [17-19] have shown good correlation and agreement between the DAT and the rK39 rapid test. Further, the DAT test for detection of Leishmania infection is no longer in use by the National Program in Bangladesh. The National VL Elimination Guideline of Bangladesh has recommended the rK39 strip test for detection of Leishmania infection in the country. The fact that none of the study participants who tested negative by the rK39 rapid test developed VL during six months of follow up indicates that the rK39 strip test did not miss Leishmania infections in our study population.

The study findings can help a guide to the National VL Elimination Program since it was carried out in Mymensingh, the highest VL endemic district in Bangladesh where the risk of VL transmission through blood transfusion would be highest.

## Conclusions

The risk for transmission of VL through donated blood from healthy blood donor volunteers in Mymensingh is very low. There is no strong justification for routine screening for Leishmania infection among blood donors in Bangladesh.

## Abbreviations

VL: Visceral leishmaniasis; KA: kala-azar; ICT: Immunochromatographic test; PKDL: Post-kala-azar dermal leishmaniasis; BTD: Blood transfusion department; HbsAg: Hepatitis B surface antigen; HIV: Human immunodeficiency virus; HCV: Hepatitis C virus; CMV: Cytomegalovirus; PCR: Polymerase chaine infection; CI: Confidence interval; FRA: Field research assistant; Ln-PCR: Nested polymerase chain reaction; DAT: Direct agglutation test.

## Competing interests

The authors declare that they have no competing interests.

## Authors’ contributions

MMH, SR, RC, APD, SKB, RH and DM conceived and designed the study, KRHB carried out the PCR analysis and helped to draft the manuscript. MMH, SR, RC, APD, SKB, RH and DM wrote the paper; MMH performed the statistical analysis; DG participated in designing the study and coordination and helped to draft the manuscript. All authors read and approved the final manuscript.

## Pre-publication history

The pre-publication history for this paper can be accessed here:

http://www.biomedcentral.com/1471-2334/13/62/prepub
